# Supplementary material for: Programmable nanocomposites of cellulose nanocrystals and zwitterionic hydrogels for soft robotics
Source: Nat Commun. 2023 Sep 30;14:6108. doi: 10.1038/s41467-023-41874-7 (PMC10542366; doi:10.1038/s41467-023-41874-7)
Supplement: Supplementary file 1 — Supplementary Information [file 41467_2023_41874_MOESM1_ESM.pdf]

Supplementary Information

Programmable Nanocomposites of Cellulose Nanocrystals and Zwitterionic Hydrogels for Soft Robotics

Rasool Nasseri[1†], Negin Bouzari[1†], Junting Huang[1], Hossein Golzar[2], Sarah Jankhani[1], Xiaowu (Shirley) Tang[2,3,4], Tizazu H. Mekonnen[1,4,5], Amirreza Aghakhani[6], and Hamed Shahsavan[1,3,4✉]

[1] Department of Chemical Engineering, [2] Department of Chemistry, [3]Centre for Bioengineering and Biotechnology, [4]Waterloo Institute for Nanotechnology, [5]Institute of Polymer Research, University of Waterloo, Waterloo, ON, N2L 3G1, Canada. [6]Institute of Biomaterials and Biomolecular Systems (IBBS), University of Stuttgart, Pfaffenwaldring 57, 70569, Stuttgart, Germany. ✉e-mail: hshahsav@uwaterloo.ca

[†] These authors made equal contributions to the work.

Supplementary Methods

Finite element simulations

The mechanical deformations of anisotropic hydrogels, shown in Fig. 5a-c, were modeled using the finite element software (COMSOL Multiphysics 6.0). The neo-Hookean hyperelastic model was used to model the swelling behavior of hydrogels, which is capable of describing the mechanical behavior of hydrogels in different regimes of large deformation. The hydrogel elastic modulus of E = 191 kPa was used for the anisotropic gels with an assumed Poisson’s ratio of 0.3, typical in most hydrogel materials, with the calculated material density of 1120 kg/m^3. Although the neo-Hookean model is not capable of modeling the anisotropic elasticity of the hyperelastic material, it can lead to correct estimation when the elastic properties of the material in different directions are close to each other. When the elastic properties of hydrogels in different directions differ drastically, anisotropic hyperelastic models such as the Ogden model or Fung model can be used. Both models are more complex than the neo-Hookean model and require more information about the material's properties.

The swelling strain was included in the total strain as $\boldsymbol{\varepsilon^T} = \boldsymbol{\varepsilon^e} + \boldsymbol{\varepsilon^s}$, where $\boldsymbol{\varepsilon^e}$ is the elastic strain tensor and $\boldsymbol{\varepsilon^s}$ is the swelling strain tensor, given by[1]:

$$\boldsymbol{\varepsilon^s} = \begin{bmatrix} \boldsymbol{\alpha}_{\parallel} & \mathbf{0} \\ \mathbf{0} & \boldsymbol{\alpha}_{\perp} \end{bmatrix}$$

where $\boldsymbol{\alpha}_{\parallel}$ and $\boldsymbol{\alpha}_{\perp}$ are swelling in parallel and perpendicular to the alignment direction (adopted from **Fig. 3d**). The swelling strains were increased by small increments in the simulations to achieve the solution convergence.

Calculation of Hermans Order Parameter

After baseline correction, the order parameter $\boldsymbol{S}$ was calculated by following equations. Here, $\boldsymbol{\gamma}$ represents the angle between the (200) lattice plane and the direction of CNC alignment and $\boldsymbol{\phi}$ is the azimuthal angle and $\boldsymbol{I(\phi)}$ is the one-dimensional intensity distribution along $\boldsymbol{\phi}$.

$$\boldsymbol{S} = \frac{3\langle\cos^2\gamma\rangle - 1}{2} \quad (1)$$

$$\langle\cos^2\gamma\rangle = 1 - 2\langle\cos^2\phi\rangle \quad (2)$$

$$\langle\cos^2\phi\rangle = \frac{\int I(\phi)\cos^2\phi\sin\phi\, d\phi}{\int I(\phi)\sin\phi\, d\phi} \quad (3)$$

Supplementary Discussion

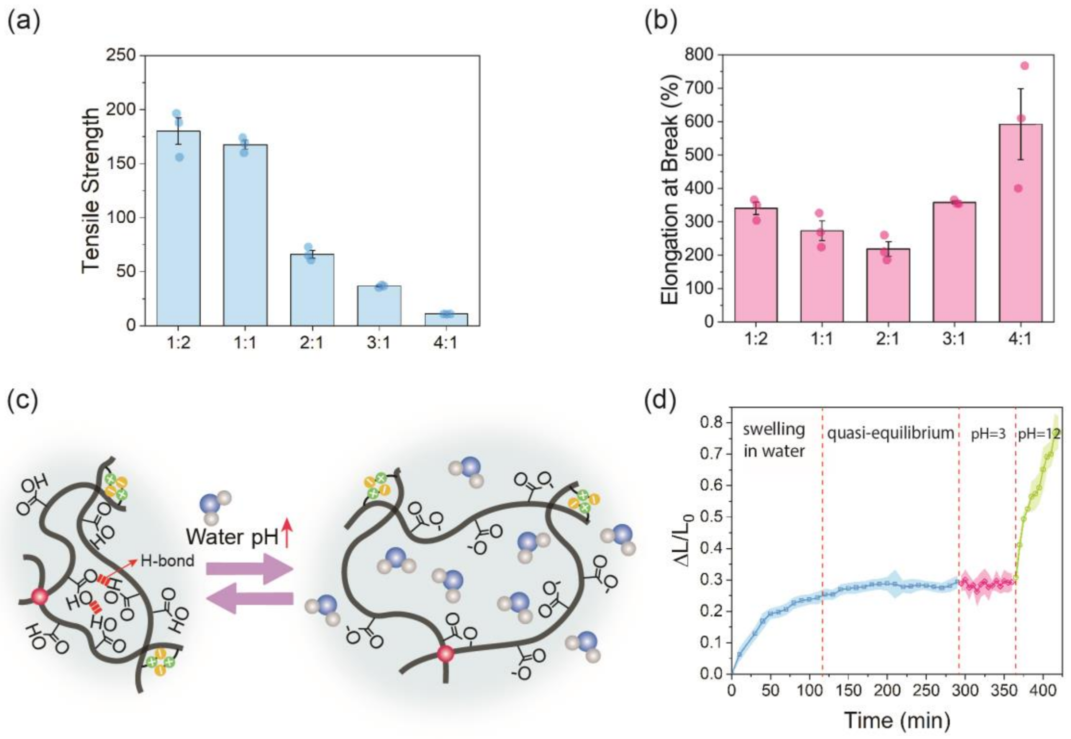

Supplementary Fig. 1. a) Tensile strength of physically-crosslinked DMAPS-MAA hydrogels containing different ratios of comonomers. The average value of at least three samples is reported. The error bars represent the standard error. b) Elongation at break of physically-crosslinked DMAPS-MAA hydrogels containing different ratios of comonomers. The average value of at least three samples is reported. The error bars represent the standard error. c) Schematic presentation of the swelling/deswelling mechanisms of a Gel sample in response to a change in pH. d) The degree of swelling of a GelWC sample in water (pH ~ 7) and in buffers with pH 3 and 12. The average value of three samples is reported. The shaded area represents the standard error.

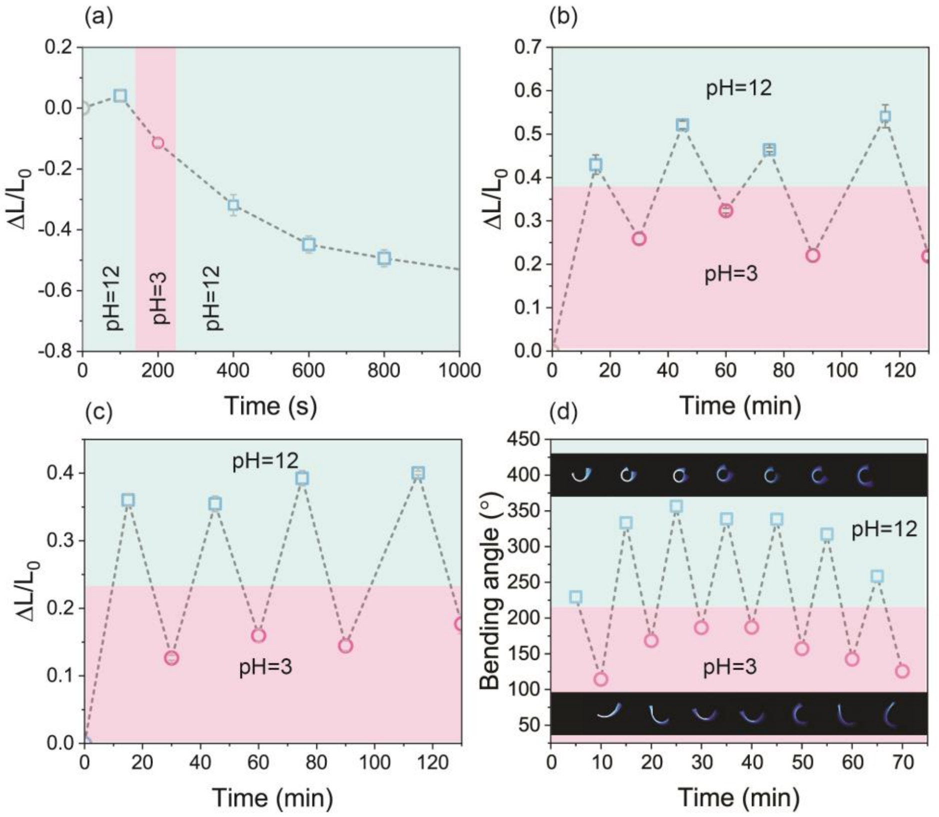

Supplementary Fig. 2. Cyclic swelling/deswelling study of a) GelWC, b) Gel, c) IGel, and d) AGel. For GelWC, Gel, and IGel the degree of swelling $(L - L_0)/L_0$ is reported and for AGel the bending angle (defined as the angle between a vertical tangent applied to one edge of the hydrogel and a vector connecting two edges of the hydrogel after bending) is reported. For GelWC, Gel, and IGel, the average value of three samples is reported. The error bars represent the standard error.

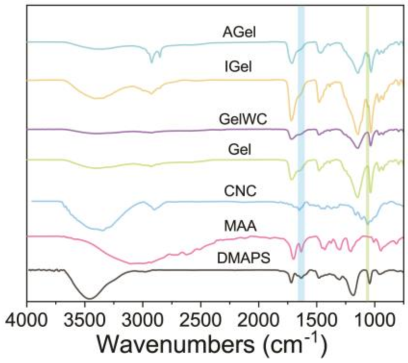

Supplementary Fig. 3. FTIR spectra of the hydrogels and their components. The C=C stretching and C-O stretching regions are highlighted in blue and green, respectively.

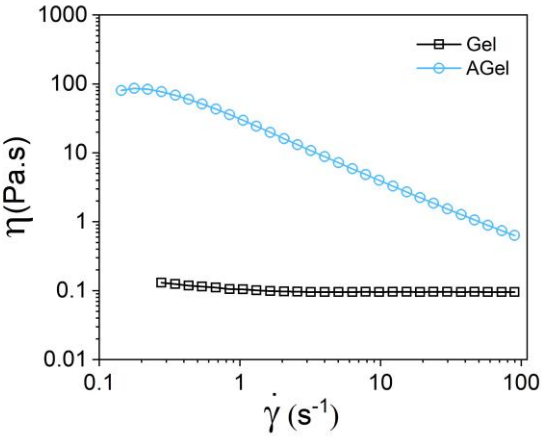

Supplementary Fig. 4. Viscosity of Gel and AGel precursors as a function of shear rate at 25 °C.

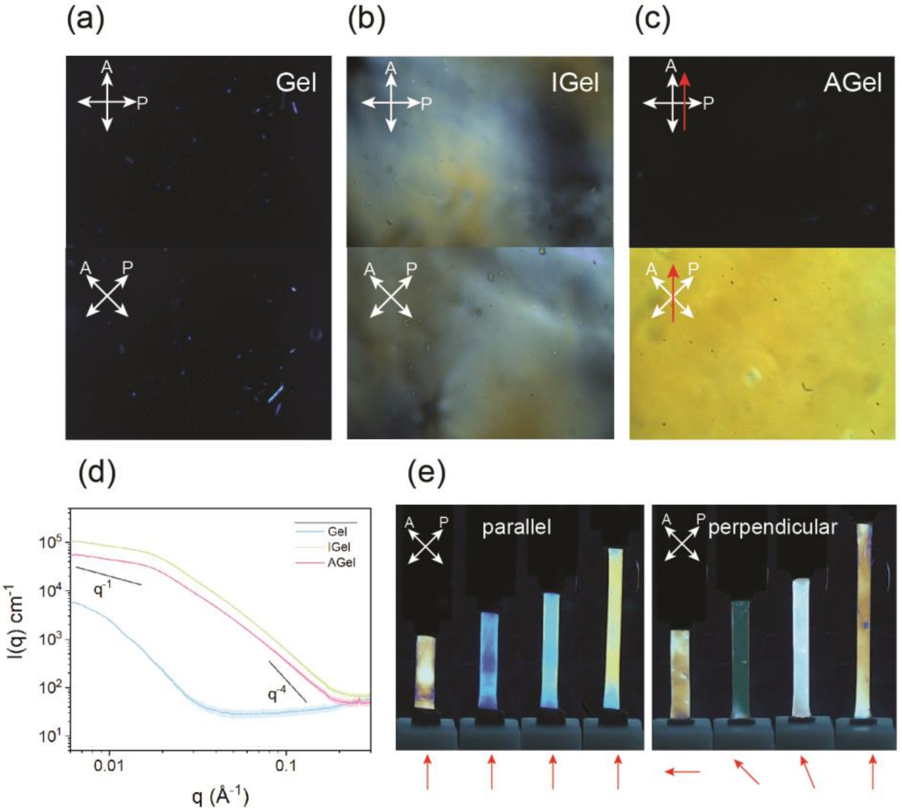

Supplementary Fig. 5. POM images of a) Gel, b) IGel, and c) AGel. d) 1D radial-averaged SAXS plots of Gel, IGel, and AGel. The low-q curves show a $\sim q^{-1}$ asymptote, which is because the length of CNC is much larger than its width. At large-q values, all profiles exhibit a $\sim q^{-4}$ asymptote, which can be explained by the presence of a sharp interface. e) The color changes of AGel samples elongated parallel to the direction of CNC alignment. CNC alignment direction was fixed at 45° relative to the polarizer and analyzer while samples were elongated parallel or perpendicular to the CNC alignment direction. CNC alignment direction is shown with red arrows.

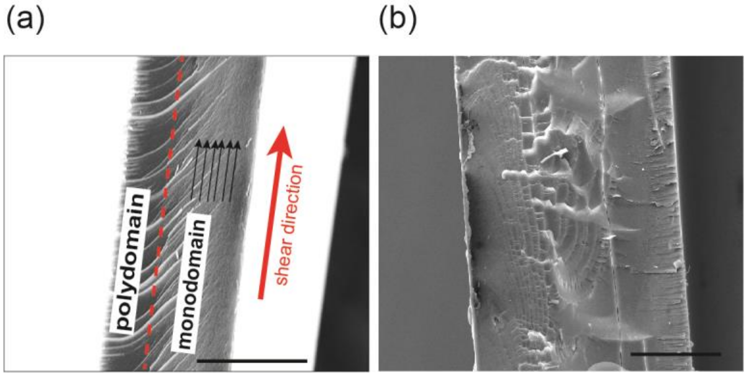

Supplementary Fig. 6 . a) SEM images of AGel cross-section with a thickness of 250 μm after drying. The scale bar is 50 μm. b) SEM images of IGel cross-section with a thickness of 500 μm after drying. The scale bar is 100 μm.

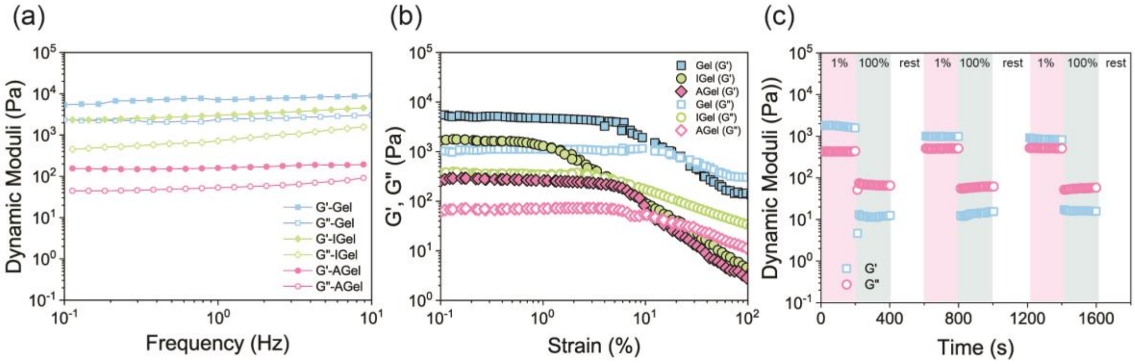

Supplementary Fig. 7. a) Frequency-dependent dynamic shear moduli of hydrogels at 1% strain and 1 Hz. b) Amplitude sweep experiment on Gel, IGel, and AGel showing the linear viscoelastic region. c) Step-strain experiment on IGel.

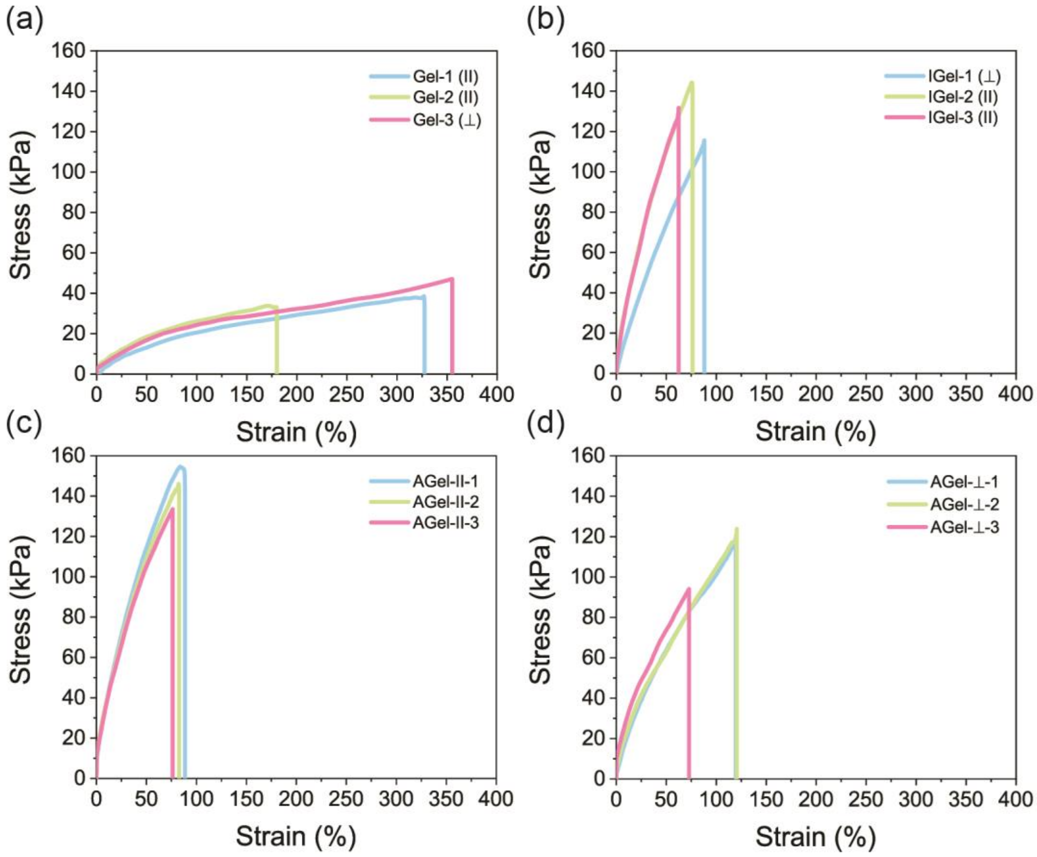

Figure S8. Tensile test repetitions on (a) Gel, (b) IGel, (c) AGel-parallel, and (d) AGel-prependicular. For Gel and IGel samples whose long axis were either parallel or perpendicular to the long axis of the hydrogels (labels show the direction of cut with respect to the long axis of the hydrogels).

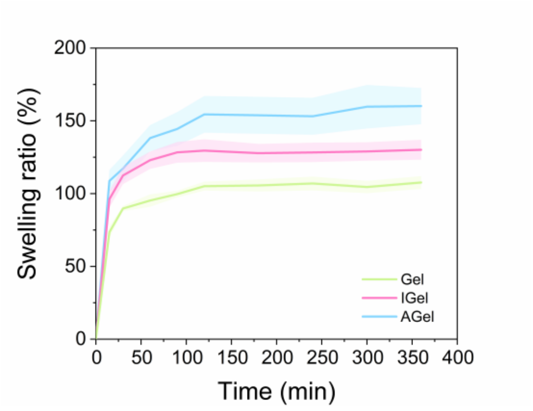

Supplementary Fig. 9. Swelling behavior of Gel, IGel, and Agel. The swelling ratio is defined as $\frac{m-m_0}{m_0} \times 100$, where m and m_0 are weight at time t and initial weight, respectively. The average value of three samples is reported. The shaded area represents the standard error. The data presented in this figure is collected by weighing the samples at different times and calculating the weight increase. Because we did not record the thickness of the hydrogel samples during the experiment, this data is not comparable with the data presented in Fig. 3g.

Generally, hydrogels containing CNCs (AGel and IGel) were swelling more due to the hydrophilicity of CNCs and their ability to absorb and maintain water. By aligning the CNCs in AGel their ability to absorb water was enhanced probably due to fewer contacts among them and more available surface area which resulted in a higher swelling ratio of AGel compared to IGel.

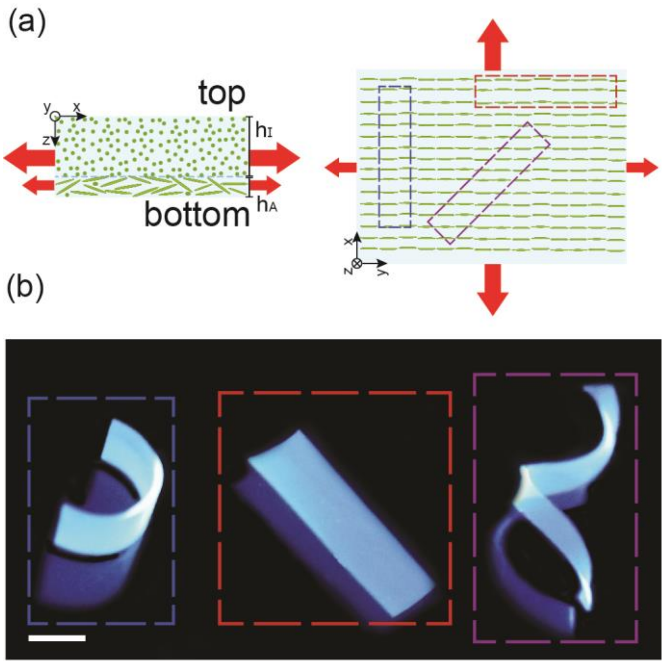

Supplementary Fig. 10. a) Schematic of CNC alignment with respect to the long axis of the cut pieces. We cut AGel film at different angles to obtain perpendicular (blue box), parallel (red box), and 45° (purple box) CNC alignment with respect to the long axis of the cut piece. Our system can be deemed as a bimorph with nonidentical swelling behavior along the thickness in which CNCs are oriented randomly on one side, with a characteristic thickness of h_I (~200 µm), and unidirectionally aligned on the other side, with a characteristic thickness of h_A (~600 µm). The size of the red arrows shows the level of swelling in different directions. b) Shape change of AGel pieces cut with the CNC alignment perpendicular to the long axis (blue box), parallel to the long axis (red box), and making a 45° angle with the long axis (purple box) in response to the change of ionic strength. The scale bars are 10mm.

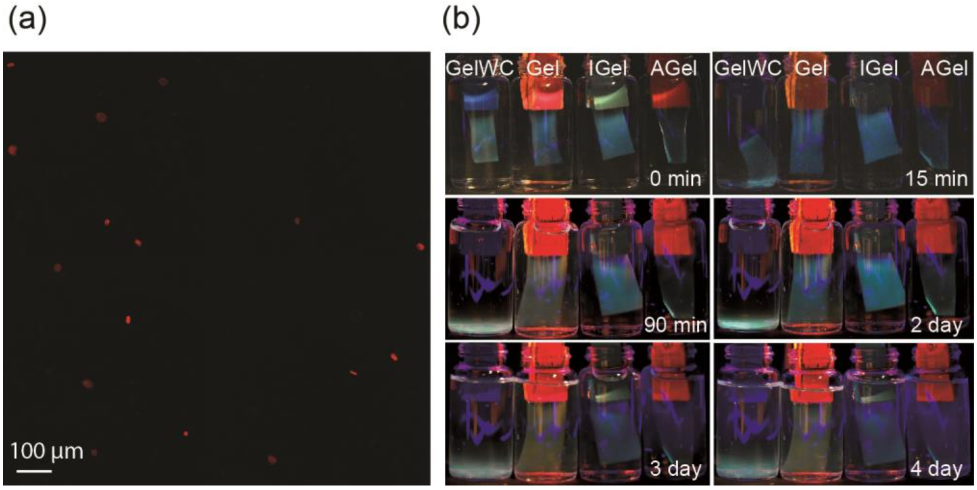

Supplementary Fig. 11. a) Confocal image of the hydrogel prepared with the filliped weight ratio of comonomers (DMAPAS:MAA weight ratio 1:3). b) Interaction of all hydrogels with 10 wt% NaCl solution for 4 days.

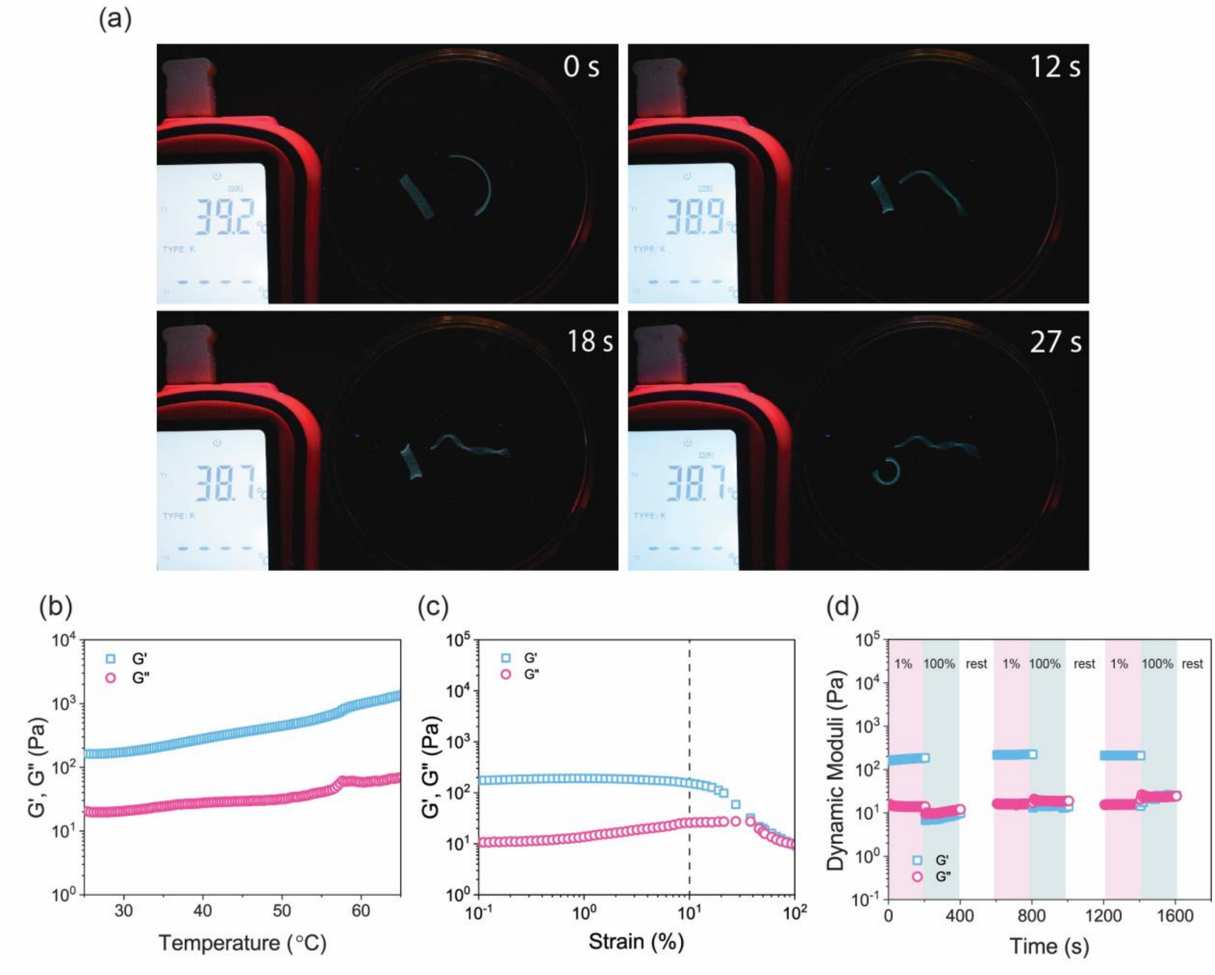

Supplementary Fig. 12. a) Shape change of AGel pieces cut with the CNC alignment perpendicular to the long axis and making a 45° angle with the long axis in response to pH change at temperatures close to the physiological temperature. b) Temperature-sweep tests on AGel. c) Strain-sweep tests on AGel at 37 °C. d) Step-strain experiment on AGel at 37 °C.

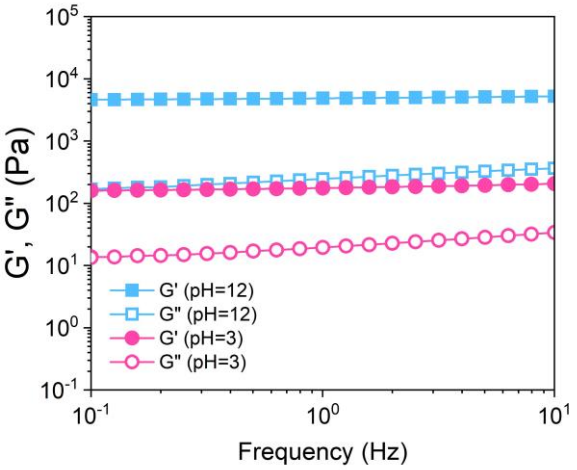

Supplementary Fig. 13. Frequency-sweep on AGel after swelling in a solution with pH of 12 and 3 for about 5 min.

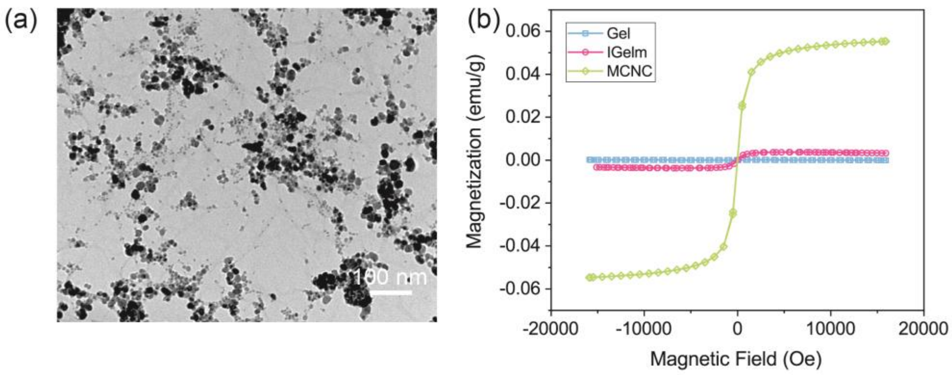

Supplementary Fig. 14. a) TEM image of MCNCs. The scale bar is 100 nm. b)VSM of Gel, IGelm, MCNC

Supplementary references

1. Sydney Gladman, A., Matsumoto, E. A., Nuzzo, R. G., Mahadevan, L. & Lewis, J. A. Biomimetic 4D printing. *Nat. Mater.* **15**, 413–418 (2016).
