# Supplementary material for: Programmable nanocomposites of cellulose nanocrystals and zwitterionic hydrogels for soft robotics
Source: Nat Commun. 2023 Sep 30;14:6108. doi: 10.1038/s41467-023-41874-7 (PMC10542366; doi:10.1038/s41467-023-41874-7)
Supplement: Supplementary file 3 — Description of Additional Supplementary Files [file 41467_2023_41874_MOESM3_ESM.pdf]

Description of Additional Supplementary Files

Supplementary Movie 1. The video shows considerable in-plane differential swelling of the AGel sample in water.

Supplementary Movie 2. The video shows elongations of a rectangle (5 mm × 25 mm) made of one piece of Gel (lower half) and one piece of AGel cut in the perpendicular direction (upper half) after 6 h of healing.

Supplementary Movie 3. The video shows the reversible bending of an AGel strip in which the alignment of CNCs is perpendicular to the long axis.

Supplementary Movie 4. The video shows the reversible bending of an AGel strip in which the alignment of CNCs is parallel to the long axis.

Supplementary Movie 5. The video shows an AGel strip in which the angle between the CNC alignment and the long axis of the strip is 45° forming a helix reversibly.

Supplementary Movie 6. The video shows complex reversible 3D shape morphing of the hydrogel construct triggered by increasing and decreasing the pH.

Supplementary Movie 7. The video shows complex reversible 3D shape morphing of the hydrogel construct triggered by increasing and decreasing the pH.

Supplementary Movie 8. The video shows complex reversible 3D shape morphing of the hydrogel construct triggered by increasing and decreasing the pH.

Supplementary Movie 9. The video shows the fast dissolution of GelWC piece in 10 wt% NaCl solution.

Supplementary Movie 10. The video shows the pH-responsive miniature gripper made by applying the cut-and-paste approach. The gripper was used to transfer light spherical cargo.

Supplementary Movie 11. The video shows the pH-responsive miniature gripper made by applying the cut-and-paste approach. The gripper was used to transfer soft biological cargo.

Supplementary Movie 12. The video shows a miniature robot that can be remotely navigated to transfer very light cargo through a confined flooded maze.

Supplementary Movie 13. The video shows the shape change of AGel pieces cut with the CNC alignment perpendicular to the long axis and making a 45° angle with the long axis in response to pH change at temperatures close to the physiological temperature.
